# Type 2 diabetes and risk of low-energy fractures in postmenopausal women: meta-analysis of observational studies

**DOI:** 10.1007/s40520-016-0562-1

**Published:** 2016-04-12

**Authors:** Joanna Dytfeld, Michał Michalak

**Affiliations:** 1 Department of Family Medicine, Poznan University of Medical Sciences, ul. Przybyszewskiego 49, 60-355 Poznań, Poland; 2 Department of Computer Science and Statistics, Poznan University of Medical Sciences, ul. Dąbrowskiego 79, 60-529 Poznań, Poland

**Keywords:** Osteoporosis, Fracture, Type 2 diabetes

## Abstract

**Background:**

Observational studies on osteoporotic fractures in patients with type 2 diabetes indicate their increased incidence compared to those without diabetes, but results are inconsistent. Currently, type 2 diabetes is not considered as an independent risk factor for low-energy fractures in elder subjects. The aim of the study was to assess the association between type 2 diabetes and risk for hip and vertebral fractures in postmenopausal women.

**Materials and methods:**

We searched Medline, Web of Science and Cochrane databases for articles published before September 2013. Studies assessing fractures in women aged >50 diagnosed with type 2 diabetes, regardless of the diabetes treatment, were deemed eligible. To estimate fracture risk meta-analysis in a random effect model was performed. The results were shown by the odds ratio (OR) and 95 % confidence interval (CI). Heterogeneity was tested using a Q-Cochrane test (significance was analyzed with *p* < 0.10) and *I* ^2^ measure.

**Results:**

A total of 15 observational studies (11 cohort and 4 cross-sectional, 263.006 diabetics and 502.115 controls) were included. Thirteen papers provided information on the incidence of hip fractures, and seven on vertebral ones. The meta-analysis revealed type 2 diabetes was associated with higher risk for hip fracture (OR 1.296, 95 % CI (1.069–1.571), but not vertebral fracture (OR = 1.134, 95 % CI (0.936–1.374). There was significant heterogeneity between hip fracture studies. American origin was identified as a potential source of such heterogeneity.

**Conclusions:**

The results of our meta-analysis indicate there is an increased risk for hip fracture in postmenopausal women with type 2 diabetes.

## Background

Diabetes and osteoporosis are two disease entities that significantly contribute to disease burden among elderly population. Prevalence of diabetes is nearly seven times higher in subjects over 60 than in the age group of 20–39 years [1]. Diabetes is estimated to affect approximately 10.9 million (ca. 26.9 %) people over 65 years of age in the USA [2], and 25–30 % of patients from that age group in Poland [3]. Owing to disease duration and associated complications, medical costs of care for diabetic patients are over two times higher as compared to healthy subjects [4].

Osteoporosis is also one of the main diseases characteristic for old age. Based on the WHO criteria, it has been diagnosed in 22 million women and 5.5 million men, aged 50–84 years, in the European Union [5]. These numbers will presumably rise considerably due to population aging. Low-energy fractures, the most significant clinical feature of osteoporosis, are often associated with severe disability and increased mortality. Available data say that the risk of death in a woman after hip fracture increases by 10-20 % when compared to her healthy counterpart [6].

Until recently, type 2 diabetes and osteoporosis have not been linked despite their co-occurrence in people over 50 years of age, especially in postmenopausal women, due to several reasons. There is no clear notion that would jointly describe bone pathology in diabetes. In type 1 diabetes absolute insulin deficiency leads to reduced bone mass density (BMD), what partly explains increased susceptibility to fractures. Meanwhile, type 2 diabetes is usually characterized by hyperinsulinemia and this difference appears to determine the quality of bone in the two subtypes of the disease. Recently, it has become apparent that patients with type 2 diabetes have increased bone mineral density [7–9]. However, many [10, 11]—but not all [12, 13]—observations in these patients report, paradoxically, an increased number of vertebral, hip, distal radius, tibia and other fractures. Therefore, the issue of low-energy fracture risk in diabetics is complex—BMD alone appears not to be the key determinant. Obesity, usually accompanying type 2 diabetes, in some ways has a protective effect on bone density, whereas low body weight has been linked to higher fracture risk [14]. Thus, determining whether type 2 diabetes contributes to increased bone fragility and should it be incorporated into the fracture risk calculator, seems to be of importance, as it may contribute to a more effective antifracture intervention.

Owing to the fact that considerable amount of new data on that topic has become available since the last published meta-analysis, we decided to answer the question whether in postmenopausal women coexistence of type 2 diabetes increases the risk of low-energy fractures, vertebral and non-vertebral, particularly of the hip.

## Materials and methods

### Search strategy

Two reviewers (JD and MM) performed the search on the association between type 2 diabetes and fracture risk independently. We limited the database search to studies carried out in humans, written in English, and published as full text before September 2013. The scope of the search included available papers from the period between 1967 and 2013. The qualified papers were assessed independently by the authors between September and November 2013.

We included observational clinical studies searched in the following medical databases: Medline (using the PubMed website), Web of Science, and Cochrane Collaboration. Potentially relevant studies were identified using the following keywords: ‘diabetes type 2’, ‘fracture risk’, ‘osteoporosis’.

### Eligibility criteria

The studies were considered eligible if they evaluated women, aged >50, diagnosed with type 2 diabetes, regardless of the treatment (diet, oral drugs or insulin). We arbitrarily assumed that the time limit of menopause would be 50 years of age. Thus, we decided not to include papers with younger subjects. If a whole study cohort was separated into age groups, we selected the one that included patients >50 years of age. Studies on men only, men and women analyzed jointly, and type 1 and 2 diabetes cases analyzed jointly were not considered for inclusion.

Diabetes was diagnosed on the basis of medical records, patient self-reports, or the fact of taking hypoglycemic medication or insulin. We wished to exclude patients with type 1 diabetes from the analysis that is why we did not analyze papers where authors incorporated subjects with diabetes from the age of 18. If possible, we analyzed disease duration and excluded papers with disease onset before the age of 18.

Studies were included only if there was a comparison group so that we could calculate odds ratios and mean differences in the outcomes between the groups. Similarly, if the control group comprised diabetics treated in a different way than the study group, such a study was also not included. When the authors of a given paper separated subgroups of type 2 diabetics into insulin and other treatments, we took into account these groups as total.

In cases where data repeated in articles published by the same authors in different journals, the work with the largest study group was taken into account.

The fractures were confirmed by medical records (reports from GPs, trained adjudicators, discharge cards), radiographs and self-reports. Vertebral and hip fractures were analyzed separately. If a study provided history of prevalent fractures and the number of fractures in prospective observation, we included data on incident fractures. Where incidence density was given, we converted it into incidence rate.

### Data extraction

Two reviewers (JD and MM) independently screened the titles for relevance. Only titles that were mutually deemed ‘irrelevant’ were excluded. Abstracts and full-text articles were then screened for inclusion/exclusion criteria. Disagreements between the reviewers were resolved by means of a discussion. Furthermore, we supplemented the electronic search by hand-searching reference lists of relevant articles and reviews. One author was contacted personally.

In case of each paper we extracted data on the surname of the first author, year of publication, country, study design, number of exposed and unexposed subjects, mean age of subjects and controls.

### Statistical analysis

The analysis was performed using the STATA software, version 13.1 (StataCorp LP, USA). Random-effects model described by DerSimonian and Laird was used to aggregate the study data. In case of zero outcome events, continuity correction was performed by adding a correction factor of 0.5. Meta-analysis was conducted in accordance with the guidelines formulated in the Cochrane Handbook for Systematic Reviews of Interventions [15]. The authors followed the Preferred Reporting Items for Systematic reviews and Meta-Analyses (PRISMA) guidelines for systematic reviews and meta-analyses. Fracture risk analysis was performed using the odds ratio (OR). Statistical heterogeneity between the studies was evaluated with the Cochrane’s Q statistics and the *I* ^2^ statistics, which demonstrated contribution of heterogeneity relative to the whole in case of each study. The significance level was established at *P* < 0.10.

The random effect model was used due to great *I* ^2^ statistics value for the analyzed studies considering both, vertebral and hip fractures. The publication bias was explored by visual inspection of funnel plots and formally—with Egger’s regression asymmetry test [16, 17]. Furthermore, sensitivity analysis was performed for parameters showing significant heterogeneity.

## Results

### Characteristics of the study group

**Fig. 1:**
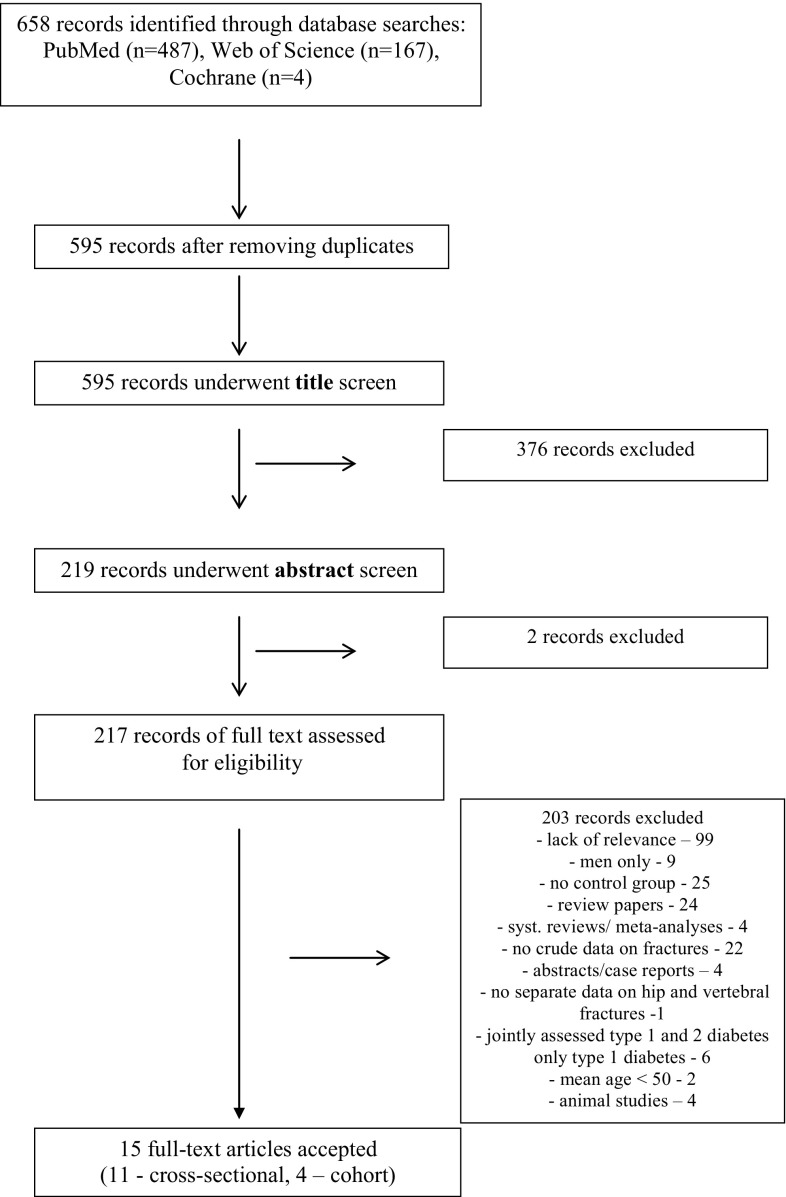
PRISMA flow diagram for inclusion of relevant studies

Figure 1 shows a flow diagram describing the study selection process. In the 15 included studies, a total of 738.121 patients were analyzed: 263.006 subjects with DMT2 and 502.115 controls (numbers without [27], as data were repeated in [28]). Patient characteristics are shown in Table 1. Out of these 15 studies, 4 studies were cross-sectional and 11 were cohort. Majority of them (except for one [20]) used incidence rate ratios as a relative risk measure; we used unadjusted measures. Five studies were conducted in the USA, 1 in Canada, 6 in Europe (Austria, Spain, Sweden, Italy), and 3 in Asia (South Korea, Japan, Taiwan). Thirteen papers provided information on the incidence of hip fractures [7, 10, 12, 13, 18–21, 24–26, 28] (in case of Schwartz et al. [27] more recent data on the same population [28] were taken into account). In seven papers there were data on vertebral ones [7, 10, 12, 21–23, 27].

**Table 1:** Characteristics of the analyzed studies

Study	DMT2			Control			Type of study	DMT2	Control	
	Hip fracture	Vertebral fracture	*n*	Hip fracture	Vertebral fracture	*n*		Mean age (years)		Region
Forsen (1999)	69	–	825	1112	–	17516	Cohort	>50	>50	Europe
Nicodemus (2001)	38	–	1682	452	–	30377	Cohort	62.3	61.5	North America
Schwartz (2001)	48^b^	23	657	501^b^	365	8997	Cohort	71.6	71.7	North America
Gerdhem (2005)	3	2	74	48	46	1058	Cross-sectional	75	75	Europe
de Liefde (2005)	28	–	483	137	–	3481	Cohort	73.8^a^	68.8^a^	Europe
Bonds (2006)	128	99	5285	1531	1336	88120	Cohort	64.9	63.5	North America
Dobnig (2006)	41	–	583	69	–	1081	Cohort	84.3	82.8	Europe
Janghorbani (2006)	125	–	8348	1255	–	101343	Cohort	61.7	55.9	North America
Lipscombe (2007)	48	–	5271	80	–	10276	Cohort	≥66	≥66	North America
Chen (2008)	8992	–	238129	5110	–	238417	Cohort	>45	>45	Asia
Sosa (2008)	1	5	111	0	5	91	Cross-sectional	71.7	69.9	Europe
Yamamoto (2009)	–	43	137	–	155	622	Cross-sectional	65.9	67.5	Asia
Schwartz (2011)	84	–	770	1117	–	8679	Cohort	73.6	73.4	North America
Gaudio (2012)	–	8	40	–	3	40	Cross-sectional	63.7	62.1	Europe
Jung (2012)	20	35	1268	12	31	1014	Cohort	60.6	61.4	Asia

DMT2—type 2 diabetes mellitus

*n*—all patients in the given category (diabetes/controls)

^a^Mean age is given for the whole study group, both men and women

^b^Hip fractures were not taken into meta-analysis since updated data were given in Schwartz 2011

### Vertebral fractures

Figure 3 presents individual study results and the overall study result for papers on vertebral fracture frequency in females with type 2 diabetes. It shows that the risk of these fractures is not different than in healthy ones OR = 1.134 (95 % CI (confidence interval) (0.936–1.374). Out of the 7 included studies, one [7] showed a difference in vertebral fracture incidence in the studied cohort when compared to the healthy females (OR = 1.240, 95 % CI (1.009–1.524). As it comprised the largest study sample, it had the greatest weight. Four studies showed lower OR when compared to controls, although the difference was not statistically significant. The range of individual risks was 0.611-3.083.

**Fig. 2:**
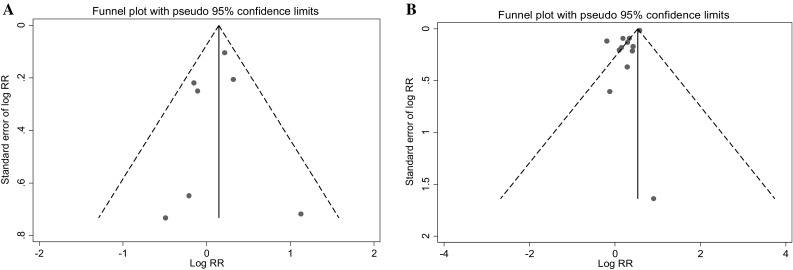
Funnel *plot* for vertebral (**a**) and hip (**b**) fracture studies

**Fig. 3:**
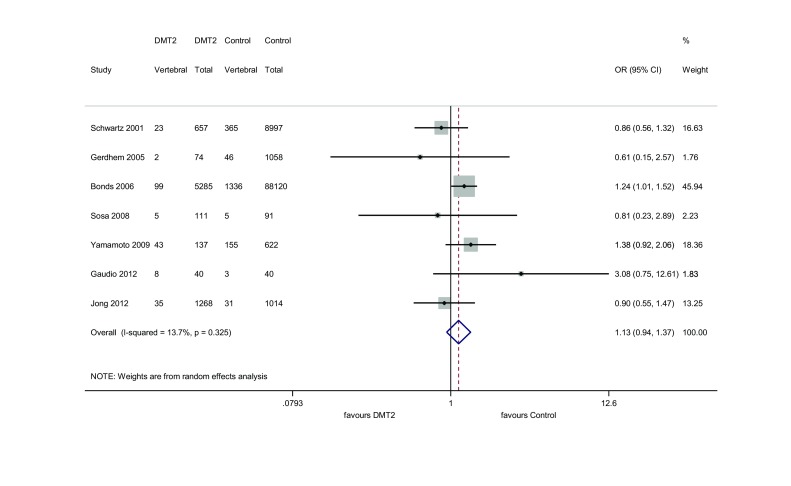
Meta-analysis for vertebral fractures

The results of the funnel plot and the Egger’s test (*P* = 0.733) show that the analysis for vertebral fractures has no publication bias (Fig. 2a). In addition, the studies appear to be homogeneous (*Q* = 6.96, *P* = 0.325, *I* ^2^ = 13.7 %) (Fig. 3).

The subgroup meta-analysis by study design shows that the summary estimate remains insignificant when it is calculated separately for cohort and cross-sectional studies (Table 2).

**Table 2:** Meta-analysis for hip/vertebral fractures by study design and geographic region

Subgroup	No. of studies	Summary OR	95 % CI	Heterogeneity		
				*Q*	*P*	*I* ^2^ (%)
Hip fracture						
Geographic area						
Asia	2	1.790	1.729–1.854	0.63	0.428	0.0
Europe	5	1.308	1.084–1.579	1.68	0.794	0.0
North America	5	1.197	0.973–1.473	14.49	0.006	72.4
Study design						
Cohort	10	1.304	1.072–1.586	74.7	0.000	87.9
Cross-sectional	2	1.007	0.330–3.075	0.35	0.556	0.0
Vertebral fracture						
Geographic area						
Asia	2	1.140	0.753–1.727	1.73	0.189	42.1
Europe	3	1.139	0.439–2.958	2.92	0.233	31.4
North America	2	1.084	0.765–1.536	2.31	0.128	56.7
Study design						
Cohort	3	1.058	0.818–1.369	3.19	0.202	37.4
Cross-sectional	4	1.315	0.889–1.944	3.11	0.376	3.4

*P*—*P* for heterogeneity

### Hip fractures

**Fig. 4:**
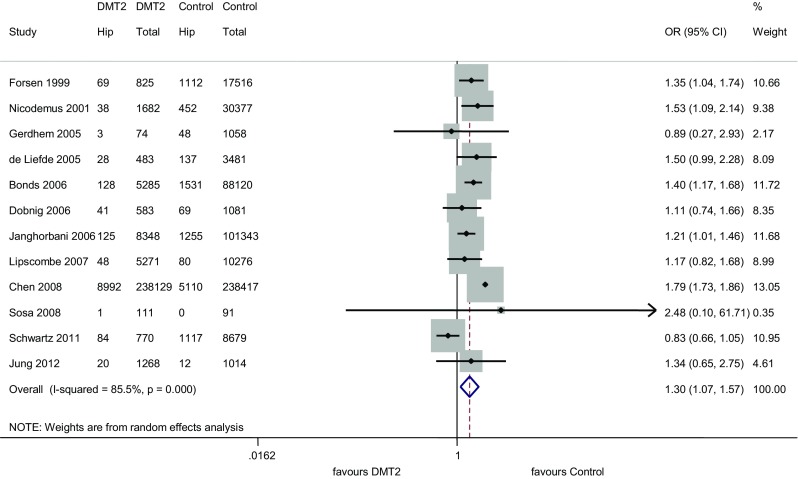
Meta-analysis for hip fractures

Out of the 12 studies on type 2 diabetes and hip fracture, five [7, 20, 24–26] found a statistically significant association and 7 found no link [10, 12, 13, 18, 19, 21, 28]. The odds ratio among the studies varied from 0.82 to 2.48 (Fig. 4). When all 12 studies were analyzed, diabetes was demonstrated to increase the risk of hip fractures by 29.6 % (95 % CI (1.069–1.571) (*P* = 0.008).

However, a significant heterogeneity between the studies (Q = 75.68, df(Q) = 11, *P* = 0.000 *p* < 0.0001, *I* ^*2*^ = 85.5 %) was observed. The sensitivity analysis showed that papers by Schwartz et al. [28] and Chen [20] contributed to heterogeneity the most. After exclusion of both papers, the association between type 2 diabetes and hip fractures was shown to increase [OR = 1.314, 95 % CI (1.193–1.448)] and the heterogeneity was no longer significant (*P* = 0.904, *I* ^*2*^ = 0,0 %).

For the hip fracture studies, the funnel plot suggested an existing bias and their importance was checked by the Egger’s test, which appeared to be significant (*P* = 0.009) (Fig. 2b).

We also conducted subgroup meta-analysis by geographical region and study design (cohort vs cross-sectional) (Table 2). The analysis revealed that the higher risk of hip fracture was attributable to cohort studies only (OR = 1.304, 95 % CI (1.072–1. 586). The cohort studies were shown to be heterogenous (*P* = 0.00; *I* ^2^ = 87.9 %). Moreover, the highest risk of hip fracture was shown in studies conducted in Asia [OR = 1.790; 95 %CI (1.729–1.854)]. For European studies the OR was 1.308 [95 % CI (1.084–1.579)].

## Discussion

The results of our meta-analysis which included 15 observational studies showed that women with type 2 diabetes aged >50 are at a higher (by ca. 30 %) risk of osteoporotic hip fracture (OR = 1.296; 95 % CI (1.069–1.571), when compared to their healthy peers. In case of vertebral fractures, diabetes did not increase the risk (OR = 1.134, 95 % CI (0.936–1.374). However, there was significant heterogeneity in case of hip fracture studies. In the subgroup meta-analysis, American origin was identified as a potential source of such heterogeneity.

Although 2 published meta-analyses revealed the risk of fracture in diabetics to be elevated by approximately 20 % [29, 30], from the scientific and clinical point of view it seems substantial to separate a particular subgroup of patients in the calculations—namely postmenopausal women. They constitute the group of the highest prevalence of osteoporosis and, owing to clinical consequences of fractures, postmenopausal women should be the actual target of antifracture intervention. At the same time, type 2 diabetes and osteoporosis often coexist in these patients, sharing some of the risk factors (immobility, age). Similarily to our observations, Vestergaard et al., showed that both men and women with type 2 diabetes are at an increased risk of hip and wrist fracture, but not spinal fracture [30]. However, the same study showed the risk of any low-energy fracture in people with diabetes of both sexes was not different than in controls. Janghorbani et al., in their meta-analysis provided data on hip fracture risk in females with type 2 diabetes and the risk ratio (RR) was 2.1 (95 % CI 1.6–2.7). RR calculated cumulatively for men and women with type 2 diabetes, regardless of age, was 1.7 (95 % CI 1.3–2.2) for hip fracture, and 1.2 (95 % CI = 1.01–1.5) for any fracture [31].

One of the studies that made heterogeneity significant in hip fracture studies was the paper by Schwartz et al., which took three observational studies into account—Study of Osteoporotic Fractures (SOF), Osteoporotic Fractures in Men (MrOS), and Health, Aging and Body Composition Study (Health ABC) [28]. Due to eligibility criteria, we did not include MrOS data. However, risk estimates were lower in this study (OR 0.829, (95 % CI = 0.655–1.049). Lower fracture incidence might have resulted from inclusion of Afro-Americans in the Health ABC study (ca. 50 % females)—there are data on lower fracture rates in Afro-American women when compared to white females [32]. This issue is more complex, as recent studies show that relative risk of non-skull fractures in Afro-American subjects with diabetes is actually higher (HR = 1.87) than in Whites with diabetes (HR = 1.22) [33]. By contrast, Schwartz et al. in separate analysis of Health ABC data did not observe an effect of type 2 diabetes on the rate of bone loss for black women at the femoral neck or total hip [34]. The role of ethnic differences in fracture incidence is open to debate. This also applies to Asian studies, which influenced hip fracture risk estimates in our meta-analysis. Given the paucity of data on low-energy fractures in Asian population, there have been no such subgroup analyses in previously published meta-analyses.

There are several complex pathways by which type 2 diabetes might influence bone health. One explanation may be the rate of bone loss or bone turnover in diabetes, which remains the topic of much heated debate. Contrary to previously cited Health ABC study, other authors demonstrated slower bone loss at the spine [35]. Lower activity of bone formation markers in patients diagnosed with diabetes was also reported [12, 36], although the data are inconsistent.

Moreover, there are reports showing that in type 2 diabetes trabecular bone structure is intact or enhanced, whereas it is the cortical bone that is preferentially compromised [37]. This finding is relevant as the cortical bone builds 80 % of the skeleton and fractures in diabetes most often occur in sites rich in cortical bone [38].

The underlying mechanisms whereby type 2 diabetes leads to increased likelihood of fracture are plentiful. Much attention has been given to advanced glycation end products (AGEs) and their receptors (RAGE), whose interaction causes pathological cross-linking in organic bone matrix, rendering the bone more fragile and brittle [39]. Urine calcium loss resulting from hyperglycemia has a detrimental effect on bone density. Negative calcium balance and—contrary to what one might expect—subsequent secondary hypoparathyroidism also play a role in the process [40]. At the same time, diabetic nephropathy itself leads to calcium/phosphorus metabolic disturbances. It is also suggested that microangiopathy contributes to impaired blood supply to the bone and that hyperglycemia worsens bone healing [41]. Besides these alterations, type 2 diabetes subjects are known to be more prone to vitamin D deficiency than their healthy peers [42]. Last but not least, falls (most often preceding fractures) are more frequent in described patients, among other things due to coexisting neuropathy, retinopathy and episodes of hypoglycemia [43].

The idea of incorporating type 2 diabetes into the fracture risk calculator seems appealing, however, according to expert opinions—premature. To evaluate clinical utility of fracture risk calculation in patients with type 2 diabetes, Schwartz et al., estimated the sensitivity of FRAX in fracture prediction in that particular group [28]. Analyzing three study cohorts (SOF, MrOs and HealthABC), they showed that the same value of a 10-year risk in women with diabetes corresponds with the *T* score of about 0.5 units higher than *T* score in healthy counterparts. In other words, women with type 2 diabetes tend to fracture at higher *T* score. That is one of the reasons why clinicians intuitively believe that DXA parameters alone do not reflect the complexity of bone pathology in diabetes. Potential mechanisms that impair bone competence and cause alterations in bone microarchitecture might be assessed by means of diverse methods (e.g. cortical thickness, trabecular bone volume, quantitative computed tomography of the bone), although the vast majority of them have not yet entered everyday clinical practice. Recently, reports on the use of bone microindentation testing have been published. Technological advancement has allowed to make this method minimally invasive [44, 45]. This technique evaluates the ability of bone to resist crack generation and propagation by measuring indentation distances performed via in vivo testing. Subsequently, bone material strength (BMS) is calculated. Studies showed BMS is affected in hip fracture patients [46].

Although our findings support the hypothesis that type 2 diabetes increases hip fracture risk, it must be considered within the context of strengths and weaknesses. One cannot exclude that patients with type 1 diabetes or LADA were taken as type 2 and vice versa—type 2 diabetes might have been undiagnosed which would, respectively, overestimate and underestimate the risk. In incorporated papers we tried to critically verify the type of diabetes in the included patients. However, the distinction between type 1 and 2 is not as clear as it was 10 or 20 years ago. We also decided to incorporate studies that relied on self-reported diabetes [28], or used old diagnostic criteria [18], which altogether might be subject to potential disease misclassification bias. Secondly, there was relatively small amount of data on vertebral fractures—which might result from underestimation of that type of fractures in general. Moreover, some papers relied on self-reports for fractures, which for vertebral ones—contrary to other types—were shown to have relatively low (51 %) validity [47]. One study [29] took all fractures—not only low-energy ones—into account.

## Conclusions

Our meta-analysis supports the association between type 2 diabetes and increased hip fracture risk in postmenopausal women. The results, however, should be interpreted with caution due to large heterogeneity of studies. Possibly, they does not answer all questions on bone fragility in patients suffering with type 2 diabetes but implies thinking about low-energy fracture probability in a growing number of postmenopausal women with coexisting type 2 diabetes but implies. Further studies are needed to elucidate the complexity of bone pathology in diabetes.
